# The Efficacy of Transversus Abdominis Plane Block for Abdominal Hysterectomy Post-operative Analgesia

**DOI:** 10.7759/cureus.3131

**Published:** 2018-08-10

**Authors:** Christina Dai, Kai Zhang, Jeffrey Huang

**Affiliations:** 1 Medicine, University of Central Florida College of Medicine , Orlando, USA; 2 Anesthesiology, University of Central Florida, Orlando, USA

**Keywords:** transversus abdominis plane block, anesthesia, nerve block, abdominal hysterectomy, tap block

## Abstract

Introduction/background

Abdominal hysterectomy is an open surgical procedure associated with considerable post-operative pain. Narcotics are often required during patient recovery but can result in adverse side effects. Transversus abdominis plane block (TAP block) is a regional anesthetic technique that is found to be an effective post-operative analgesia for many types of abdominal surgeries, including abdominal hysterectomies. However recent literature shows contradicting results regarding TAP block’s analgesic effect on recovery from abdominal hysterectomies. This study investigated the efficacy of TAP block to reduce narcotic consumption and improve pain scores in abdominal hysterectomy patients.

Methods

A single-center retrospective cohort study was performed. The inclusion criteria was patients who underwent abdominal hysterectomy at Winnie Palmer Hospital for Women & Babies (WPH) between January 12, 2015 and December 31, 2015. Exclusion criteria were patients who received a second surgery within the same hospitalization, experienced an in-hospital mortality event, received hysterectomy for known malignancy, stayed in the hospital less than 24 hours, and whose charts contained missing data points.

Data collected were: age, weight, height, body mass index (BMI), length of hospital stay, total narcotic consumption (intra-operation, in the post-anesthesia care unit (PACU), first 24 hours after admittance, during entire hospital stay, total patient-controlled analgesia (PCA) quantity, and total oral narcotics quantity. All narcotics were converted into parental morphine units for analysis. Numeric pain rating scale (NPRS) scores at two, four, eight, 12, 16, 20, and 24 hours after leaving the PACU were collected. Two-tailed paired T-test was performed to compare the narcotic consumption and pain scores between the TAP block group and the non-TAP block group.

Results

WPH used ultrasound-guided bilateral TAP block for patients undergoing abdominal hysterectomies. 63 patient charts were evaluated with 32 in the TAP block group and 31 in the non-TAP block group. Narcotic consumption was significantly different in the PACU with TAP block group consuming less narcotics than non-TAP block group (5.05 vs 8.65 IV morphine equivalents, *p*=0.012). TAP block group’s mean narcotic consumption was not significantly lower than Non-TAP block group’s mean consumption during intra-operation, first 24 hours after admittance, and total hospital stay (*p*=0.419, *p*=0.533, *p*=0.754 respectively). Mean NPRS scores at all hours (2, 4, 8, 12, 16, 20, and 24) displayed no statistical difference between the two groups. Total patient-controlled analgesia (PCA) and total overall oral narcotic usage showed no statistically significant differences between TAP block group and Non-TAP block group (*p*=0.252, 0.669 respectively).

Conclusion

The results of this study demonstrated that TAP block did reduce narcotic requirement in the PACU but did not exhibit superior analgesic efficacy after discharge from the PACU, nor reduce the total length of hospital stay.

## Introduction

Abdominal hysterectomy is a common surgical procedure associated with considerable post-operative pain [1]. The use of transverse lower abdominal incisions in abdominal hysterectomies and myomectomies often cause severe pain during the first 48 hours postoperatively which can adversely impact healing, patient outcomes, and prolong the length of hospital stay [2]. Narcotics such as morphine are often required during patient recovery but can result in adverse side effects such as sedation, pruritus, and nausea and vomiting [1, 3-4]. Therefore, alternative analgesic methods need to be considered and explored.

First introduced in 2001, transversus abdominis plane (TAP) blocks are a widely used regional anesthetic that introduces local anesthetics into the neurovascular plane via the ‘Triangle of Petit’ [5]. The Triangle of Petit, also known as the inferior lumbar triangle, was first described by the French surgeon Jean Louis Petit in 1774 as a weakened area of dorsal abdominal wall bordered by the iliac crest inferiorly, the medial edge of the external abdominal oblique laterally, and the lateral edge of the latissimus dorsi medially [6]. This procedure blocks the sensory nerves of the anterolateral abdominal wall, T6-L1, that travel to innervate the abdomen [7]. It has been shown to provide effective analgesia for laparoscopic surgeries [8] and for numerous other abdominal procedures [9]. 

The effectiveness of this procedure as a post-operative analgesic option for patients undergoing abdominal hysterectomies has been controversial as recent studies have found mixed results on its analgesic benefits in gynecological procedures [10-11]. Therefore, the primary objective of this study is to quantify and compare the efficacy of TAP block as a post-operative analgesia for patients who underwent abdominal hysterectomies versus standard post-operative pain management. We hypothesized that patients who received the TAP block will have significantly lower narcotic consumption and self-reported numeric pain rating scale (NPRS) scores for post-operative pain compared to the patients who did not.

## Materials and methods

This was a retrospective cohort study conducted at Winne Palmer Hospital for Women and Babies in Orlando, Florida. After institutional review board approval, medical records of all patients with abdominal hysterectomies between January 1, 2015 and December 31, 2015 were reviewed. Exclusion criteria for the study were: patients who received a second surgery within the same hospitalization, experienced an in-hospital mortality event, received hysterectomy for known malignancy, received laparoscopic hysterectomy, stayed in the hospital less than 24 hours, and whose charts contained missing data points.

All patients in the TAP block group received bilateral ultrasound-guided TAP blocks following the hysterectomy. With each block, between 20cc 0.25% PF bupivacaine to 60cc 0.25% PF bupivacaine was administered. The non-TAP block group received standard narcotics as needed for pain control.

Statistics

Data collected included participant baseline characteristics: age, weight, height, body mass index (BMI). Length of hospital stay, time in post-anesthesia care unit (PACU), and duration of surgery were also collected. These are reported descriptively as a mean ± standard deviations and compared using a two-tailed T-test.

The primary endpoint of pain control was measured both subjectively through numeric pain rating scale (NPRS) scores and objectively through total narcotic consumption. Total narcotic consumption was measured at four different time intervals during each patient’s stay: during surgery, in the PACU, first 24 hours after leaving the PACU, and from admission to discharge. Quantities of oral and parenteral narcotic use were converted into parenteral morphine units for analysis [12]. NPRS scores were collected 2, 4, 8, 12, 16, 20, and 24 hours after leaving the PACU. These are reported as means and a two-tailed T-test was performed to compare the differences between the two groups with a *p *<.05 used for statistical significance. Statistical analyses were conducted using the Statistical Package for the Social Sciences (SPSS v.24.0: IBM; Chicago, IL) and Excel.

## Results

A total of 63 patients were evaluated for this study: 32 received the TAP block post-operatively and 31 received conventional post-operative management. Except for age, the two groups possessed comparable baseline characteristics as the TAP block group was slightly older than the group receiving standard pain management (49 years old vs 45 years old, p=0.035) (Table 1). The length of hospital stay, time in the PACU, and operative time were also similar as there was no significant difference between the two groups.

**Table 1:** Baseline Characteristics and Outcomes of Study Groups sd: standard deviation, cm: centimeter, kg: kilogram.

Demographics and Outcome Variables	TAP- Block [mean (sd)]	No TAP block [mean (sd)]	P-value
Age (years)	49 (10)	45 (6)	0.035
Height (cm)	161.77(7.50)	162.97 (7.03)	0.521
Weight (kg)	81.95(18.40)	78.84(14.40)	0.465
BMI	31.20 (6.09)	29.75 (5.62)	0.331
Length of Hospital Stay (days)	2.87 (1.31)	2.60 (0.74)	0.336
Time in PACU (minutes)	114.19 (6.75)	151.32 (51.84)	0.535
Surgical Time (minutes)	115.38 (46.21)	120.00 (53.72)	0.715

**Figure 1:**
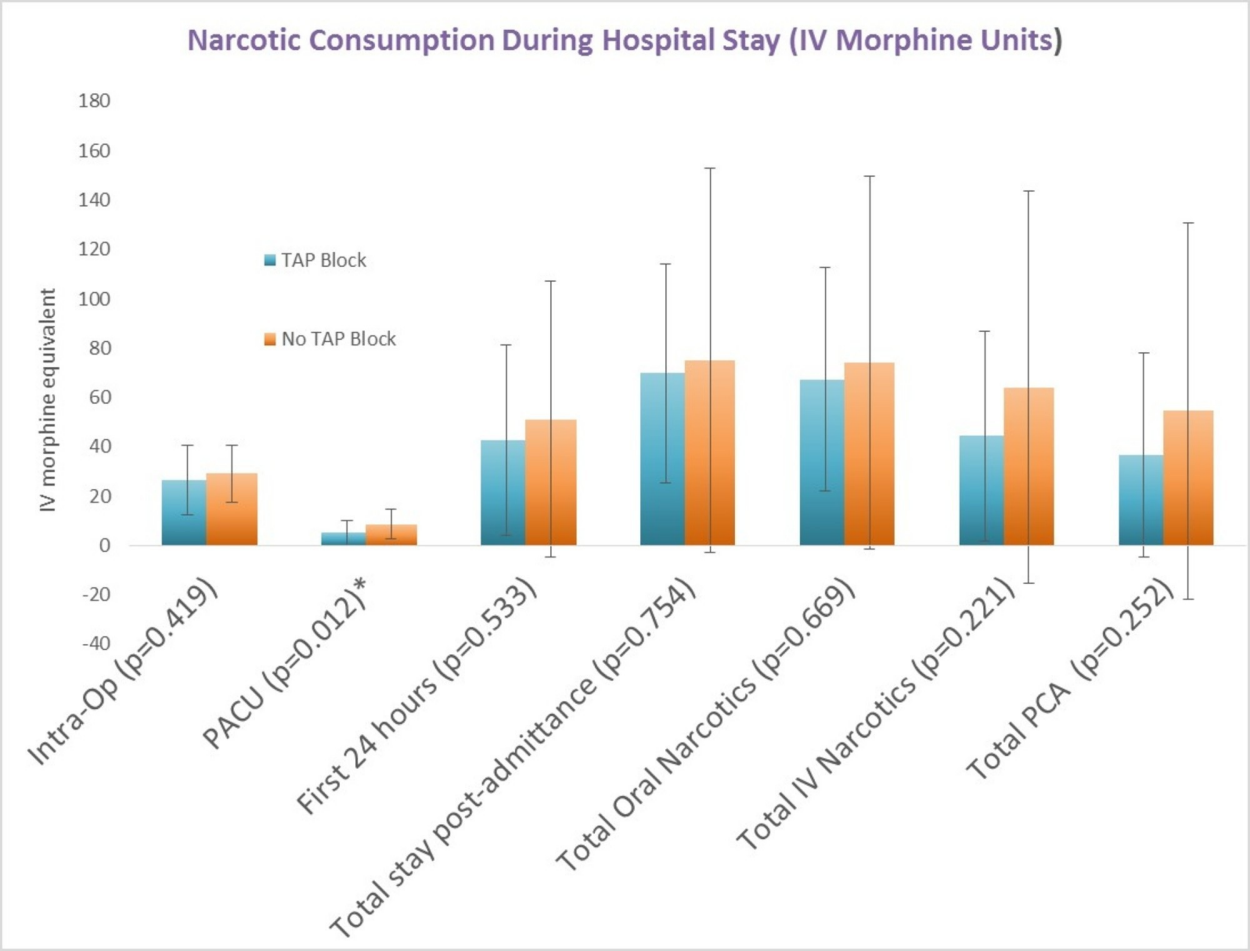
Narcotic Consumption During Hospital Stay PACU: post-anesthesia care unit, PCA: patient-controlled analgesia.

Figure 1 demonstrates that the amount of narcotic consumption in the TAP block group was significantly less compared to non-TAP block group while in the PACU (5.05 vs 8.65 IV morphine equivalents, *p *= 0.012). Although mean narcotic consumption was also less in the TAP group at other time points such as intra-operatively, first 24 hours post admission, and total hospital stay, this difference was not found to be statistically significant (*p*=0.419, *p*=0.533, *p*=0.754 respectively). In addition, total patient-controlled analgesia (PCA) and total overall narcotic usage also demonstrate no statistically significant difference between the TAP block group and non-TAP block group (*p*=0.252, 0.669).

**Figure 2:**
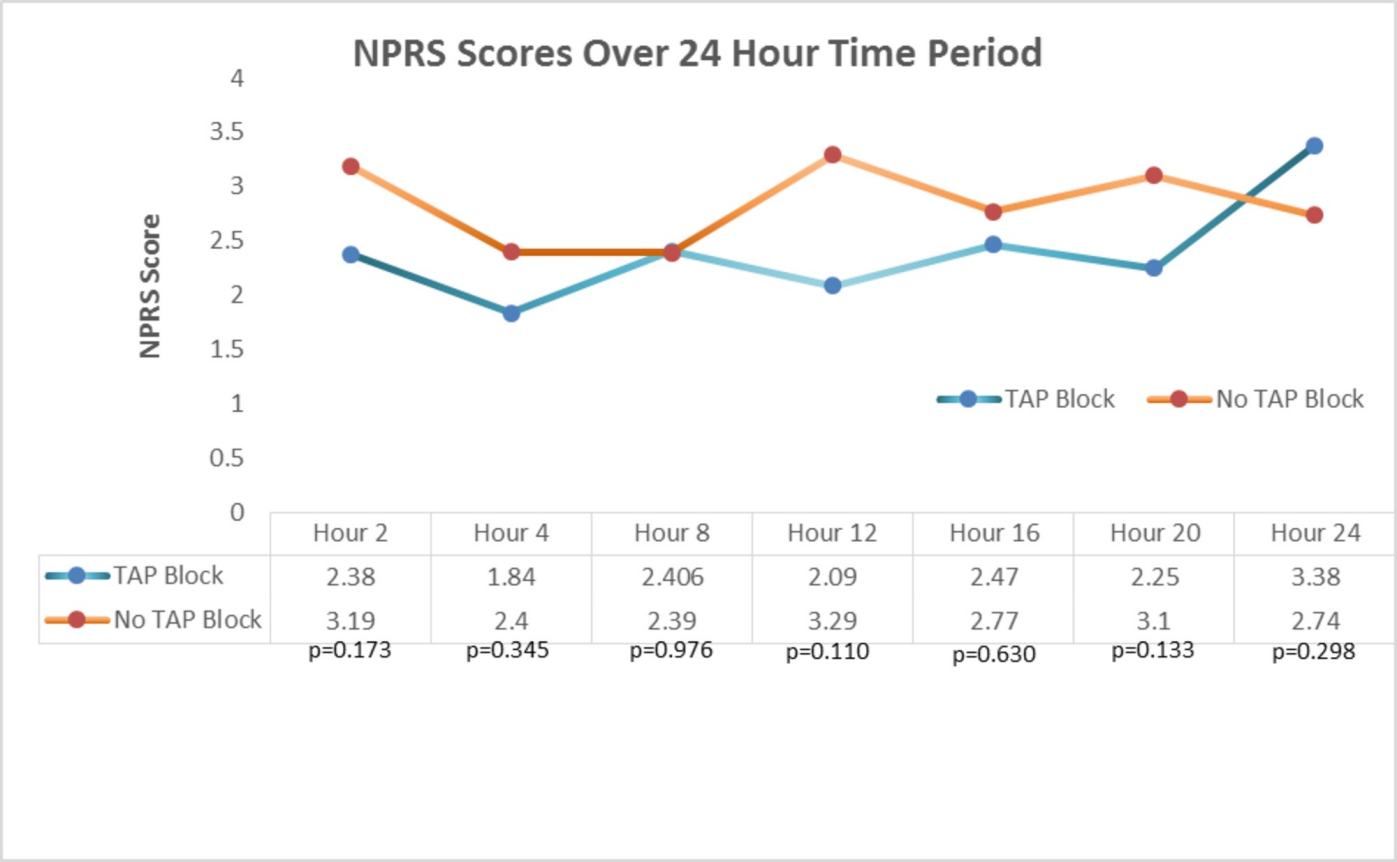
NPRS Scores Over 24-Hour Time Period TAP Block: transverse abdominis plane block, NPRS: numeric pain rating scale.

The mean NPRS scores revealed no significant difference in postoperative pain at any time point (2, 4, 8, 12, 16, 20, and 24 hours) between the two groups (Figure 2).

## Discussion

The findings of this study suggest that the TAP block did not provide superior analgesic efficacy for post-abdominal hysterectomy pain. While the TAP block did reduce narcotic requirements in the PACU, it failed to do so at other time points in the study and failed to reduce total hospitalization time. Since the TAP blocks were administered either immediately post-operation or in the PACU, the significant difference in narcotic consumption between the TAP block group and the non-TAP block group may indicate that the TAP block is effective only immediately after administration. This may be explained by the short duration of action of bupivacaine. At all other time points, narcotic consumption was less in the TAP block group, but this difference was not statistically different. This marginal reduction in opioid consumption may not translate to meaningful long-term outcomes such as narcotic-associated adverse effects and shorter hospitalization time. This study failed to demonstrate the TAP block’s ability to significantly relieve postoperative pain in our patients, but we believe that there are modifications that can be made to our TAP blocks which may yield better analgesic effects. Since we did find the TAP block to be effective in the period immediately following surgery, perhaps using a longer acting analgesic agent such as liposomal bupivacaine may provide more effective pain relief as recent studies have demonstrated that this formulation can provide longer pain control compared to traditional bupivacaine [13]. Employing a continuous TAP block through a catheter may also extend the duration of the block as some studies suggest this is a superior technique to a single shot block [14-15]. Last, the timing of administration of the block may have a role in its efficacy as indicated by some studies. A meta-analysis by Bacal et al found that pain relief appeared to be more effective when the TAP block was performed preoperatively prior to abdominal incision rather than following postoperatively as in our study [16].

The NPRS score also failed to show an improvement in pain management with the TAP block group for the first 24 hours after leaving the PACU. De Oliveira et Al suggests that patients who are in greater pain consume higher quantities of narcotics, thus leveling out pain scores between the two groups [8]. The early pain scores at hours 2 and 4 were slightly lower in the TAP block group. We speculate that potential confounding factors may have diminished the difference between in pain relief. One notable factor is the mean age of the TAP block group was four years older than the non-TAP block group which could indicate more associated comorbidities or more severe disease leading to more postoperative pain. However, the sample is relatively small, and likely contributed to the statistical difference in age.

The effectiveness of the TAP block as a post-operative analgesic option for abdominal hysterectomies remains controversial as demonstrated by our study. While Carney et al. observed a significant reduction in morphine consumption in patients who underwent an elective TAP block other studies have been unable to reproduce its findings [10-11, 17]. In addition, there has been dispute over TAP block’s superiority as a post-hysterectomy analgesic compared to other methods. Atim et al. observed ultrasound-guided TAP block to be a more effective analgesic than surgical site infiltration while Gasanova et al found surgical site infiltration to be a far superior analgesic [18]. Possible explanations for the lack of TAP block’s effectiveness can include anatomic variations which can prevent the spread of local anesthetics and the variable segmental origin of nerves in the anterior abdominal wall which may limit the usefulness of TAP block in lower abdominal procedures [19]. Furthermore, TAP blocks have been shown to be effective at controlling parietal pain which is pain from the skin and muscles from the surgical incision; however, it is not effective in reducing the visceral pain from intraabdominal structures [20].

These results of this study should be considered under the context of its limitations. This is a retrospective study with a small sample size causing a difference in age between groups as well as a large standard deviation for measured data. Other limitations include being a single institution study, the heterogeneity of the patients in this study, and the lack of measured American Society of Anesthesiologists (ASA) scores. Although we attempted to control for potential confounding factors such as preexisting malignancy and severity of the -patients' illness, we could not control for all pre-existing conditions and additional procedures performed during the same operation. We can address some of these limitations by reproducing this in a prospective study over multiple hospital sites.

## Conclusions

The results of this study demonstrated that TAP block did reduce narcotic requirement in the PACU but did not exhibit superior analgesic efficacy overall, nor reduce the length of hospital stay. These results suggest that TAP block may not be an effective strategy in improving analgesic outcomes of patients undergoing abdominal hysterectomy.
